# Silencing of IL13RA2 promotes partial epithelial‐mesenchymal transition in hepatocellular carcinoma via ERK signaling pathway activation

**DOI:** 10.1002/2211-5463.12774

**Published:** 2020-01-10

**Authors:** Mimi Wang, Rongrong Yao, Yanhong Wang

**Affiliations:** ^1^ Liver Cancer Institute Zhongshan Hospital Fudan University Shanghai China; ^2^ Huashan Hospital Fudan University Shanghai China

**Keywords:** epithelial‐mesenchymal transition, ERK signaling, hepatocellular carcinoma, interleukin‐13 receptor alpha 2

## Abstract

Lack of insight into the mechanisms underlying hepatocellular carcinoma (HCC) metastasis has hindered the development of curative treatments. Overexpression of interleukin‐13 receptor alpha 2 (IL13RA2) has been reported to contribute to invasion and metastasis in several tumors. However, the role of IL13RA2 in HCC remains to be characterized. In this study, we identified that low expression of IL13RA2 is associated with poor survival of patients with HCC, and demonstrated that IL13RA2 knockdown endows HCC cells with invasive potential. Mechanistically, silencing of IL13RA2 promotes partial epithelial‐mesenchymal transition via increasing extracellular signal‐regulated kinase phosphorylation in HCC. Collectively, our results suggest that IL13RA2 may have potential as a prognostic biomarker for HCC.

## Graphical abstract

Expression of interleukin‐13 receptor alpha 2 (IL13RA2), one of the receptors for interleukin‐13, is low in hepatocellular carcinoma (HCC) compared with normal hepatic tissue. Silencing of IL13RA2 promoted tumor cell migration via intrinsic phosphorylation of extracellular signal‐regulated kinase, and The Cancer Genome Atlas analysis suggests that IL13RA2 may be a potential prognostic biomarker of long‐term survival for patients with HCC.

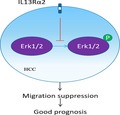

**Abbreviations**
CCK‐8: Cell Counting Kit‐8
DMEM: Dulbecco’s modified Eagle’s medium
EGFRvIII: epidermal growth factor receptor variant III
EMT: epithelial‐mesenchymal transition
ERK: extracellular signal‐regulated kinase
GEO: Gene Expression Omnibus
HCC: hepatocellular carcinoma
IL13RA2: interleukin‐13 receptor alpha 2
OS: overall survival
SD: standard deviation
TCGA: The Cancer Genome Atlas

Up to 2018, hepatocellular carcinoma (HCC) was evaluated as the sixth most common cancer in the world and the fourth leading cause of cancer death 1. With the advancement of diagnostics and the popularity of surveillance programs, the proportion of early‐stage HCC in the diagnosis of HCC has increased significantly. In developed countries, 40% of patients with HCC could be diagnosed as early stage, for which curative treatment is available 2, 3, 4. Nonetheless, the metastasis and recurrence of HCC are a major obstacle to improving overall survival (OS) and quality of life of patients. However, the mechanisms underlying the metastasis of HCC remain obscure, leading to limited metastasis‐targeted therapeutics.

Metastasis is a dynamic procedure that includes invasion to surrounding matrix, intravasation, survival and transit in the circulating system, extravasation, seeding in distant organs and clonal proliferation to form a metastasis niche. Genetic and epigenetic changes that form networks or pathways are involved in the whole metastatic process. Therefore, understanding deregulated gene expression that drives metastasis initiation is of great importance, both for prognosis and for therapeutic targets.

Interleukin‐13 receptor alpha 2 (IL13RA2), 43 kDa, is one of the receptors for IL‐13 5. IL‐13 is a T helper 2 (Th2) molecule involved in inflammation, wound healing, allergy and immune regulation. Moreover, IL‐13 plays a key role in many pathological processes, such as asthma, pulmonary fibrosis and ulcerative colitis 6, 7, 8. The classic pathway for IL‐13 activation is JAK/STAT6 via binding to receptor interleukin‐13 receptor alpha 1, not IL13RA2. IL13RA2 had once been deemed as a decoy receptor 9, 10, 11, but recent studies have demonstrated that IL‐13 could act on IL13RA2 to activate extracellular signal‐regulated kinase (ERK)/activator protein 1 12, 13 and Scr/phosphoinositide 3‐kinase/Akt/mammalian target of rapamycin 14 downstream pathways. Up to now, we concluded that IL13RA2 has two forms: transmembrane form and extracellular soluble form 15, 16, 17. The former is involved in signaling of ligands such as IL‐13, as well as binding to other membrane receptors, such as epidermal growth factor receptor variant III (EGFRvIII) 18, to form different functional subunits. As a decoy receptor, soluble IL13RA2 appears to inhibit the effects of IL13 7, 15. Taken together, the functions of these two isoforms could be antagonistic.

In several solid tumors, studies reported that IL13RA2 could predict poorer survival, such as glioblastoma 18, breast cancer 19, colorectal cancer 20 and pancreatic cancer 21. In addition, these studies revealed that overexpression of IL13RA2 could confer invasive and metastatic ability on tumor cells. However, the roles of IL13RA2 on HCC are poorly understood. In this study, we aimed to detect the expression level of IL13RA2 on HCC and to evaluate its contribution to metastasis.

## Materials and methods

### Cell culture

The human HCC cell lines—Hep G2, PLC/PRF/5, SMMC7721, Huh7, MHCC97L, MHCC97H, HCCLM3 cell line and the hepatocyte cell line L02—were all obtained from the Liver Cancer Institute at Fudan University in Shanghai, China. Among them, MHCC97L, MHCC97H and HCCLM3 are all of high metastatic potential, deriving from patent MHCC97 cells; MHCC97L has a relatively low metastatic potential, whereas MHCC97H and HCCLM3 have relatively high metastatic potential. All cells were cultured in Dulbecco’s modified Eagle’s medium (DMEM) containing 10% FBS (CellSera, Rutherford, NSW, Australia) in an atmosphere of 5% CO_2_ at 37 °C.

### Lentivirus short hairpin RNA transfection

Short hairpin RNA was used to construct recombinant lentivirus. The sequence to target human *IL13RA2* was as follows: 5′‐UCAGGAUAUGGAUUGCGUA‐3′; and the vector was PGMLV‐hU6‐MCS‐CMV‐ZsGreen1‐PGK‐Puro‐WPRE. The whole products were designed by Genomeditech Company (Shanghai, China), and HCC cell lines were transfected with lentivirus following the manufacturer’s protocol.

### Real‐time quantitative PCR analysis

Total RNA was extracted from cells using TRIzol Reagent (Sigma, Saint Louis, MO, USA), and the cDNA was synthesized with PrimeScript RT reagent kit with gDNA eraser (Takara, Kusatsu, Japan). Quantitative real‐time PCR was performed on CFX96 Touch (Bio‐Rad Laboratories, Hercules, CA, USA) using SYBR premix ex Taq (TliRNaseH Plus) (Takara). The specific primer pairs used for human *IL13RA2* are as follows: forward 5′‑ACCTGGCATAGGTGTACTTCT‑3′ and reverse 5′‑CCAAATAGGGAAATCTGCATCCT‑3′. Glyceraldehyde‐3 phosphate dehydrogenase (GAPDH) was used as endogenous control.

### Western blot analysis

Cells were lysed in radioimmunoprecipitation assay buffer with 1% PMSF and 10% phosphatase inhibitor (Beyotime Biotechnology, Shanghai, China), the protein concentrations were measured using the bicinchoninic acid method (Beyotime Biotechnology), and 20 µg protein per sample was separated in 10% SDS/PAGE using electrophoresis and transferred to polyvinylidene fluoride membranes (Millipore, Billerica, MA, USA). After 2 h of blocking with a TBST buffer (Sangon Biotech, Shanghai, China), containing 5% fat‐free milk at room temperature, the membranes were incubated with primary antibodies at 4° C overnight. The next day, the membranes were washed three times with 1× TBST and then incubated with corresponding secondary antibodies for 1 h at room temperature. Finally, the blots were detected by Immobilon™ Western Chemiluminescent HRP Substrate (ECL; Millipore). The primary antibodies we used in the research are listed as follows: IL‐13 Rα2 (#AF146; R&D Systems, Minneapolis, MN, USA), E‐cadherin (#ab40772; Abcam, Cambridge, MA, USA), N‐cadherin (#ab76011; Abcam), Vimentin (#ab92547; Abcam), Erk 1/2 (#9102; Cell Signaling Technology (CST), Danvers, MA, USA), phospho (p)‐Erk 1/2 (#4370; CST) and GAPDH (#AF0006; Beyotime Biotechnology).

### Flow cytometry analysis of apoptosis

Cells were collected, resuspended into 1 × 10^6^/mL in 200 µL 1× binding buffer and added to 5 µL Annexin V Recom APC and 5 µL 7‐aminoactinomycin D (BD Pharmingen, San Diego, CA, USA). After incubation at room temperature for 15 min away from light, cells were added to 300 µL 1× binding buffer to be analyzed in a flow cytometer (FACSCalibur; BD Biosciences, San Jose, CA, USA).

### Cell proliferation assay

Four thousand cells per well were seeded into a 96‐well plate. Cell proliferation assay was performed using Cell Counting Kit‐8 (CCK‐8; Beyotime Biotechnology) 24, 48, 72 and 96 h after cell seeding. The absorbance was detected at a wavelength of 450 nm (*A*_450 nm_) after 110 µL mixture reagent (10 µL CCK‐8 and 100 µL DMEM) was added into each well and incubated at 37° C for 1 h.

### Wound‐healing assay

We used a 200‐µL plastic pipette tip to make a wound in a six‐well plate with a cell density of 90% confluence. Subsequently, the cell was washed gently twice with PBS and cultured with DMEM containing 5% FBS. Scratches were photographed at 0, 24, 48 and 72 h after wounding. We used imagej (National Institutes of Health, Bethesda, MD, USA) to analyze the area of wound healing in different time points: Wound‐healing percentage = (scratch area in 0 h − scratch area in 48 or 72 h)/scratch area in 0 h.

### Cell migration assays

A total of 1 × 10^5^ cells with 100 µL FBS‐free DMEM were plated in the upper chamber (8.0‐μm pore size; Corning Incorporated, Corning, NY, USA), and 600 µL 10% FBS DMEM was added into the lower chamber. After 72 h, migrating cells were fixed by 4% paraformaldehyde (Beyotime Biotechnology), stained with crystal violet (Beyotime Biotechnology) for 20 min and photographed in a high‐magnification field of vision.

### Statistical analysis

Kaplan–Meier survival analysis in Kaplan–Meier Plotter used the log rank test. Gene Expression Profiling Interactive Analysis (http://gepia.cancer-pku.cn/) 22 was used to determine whether one gene is expressed differently between HCC and normal hepatic tissue in The Cancer Genome Atlas (TCGA) database, where the *P* value was calculated using one‐way ANOVA; the cutoff of |log_2_FC| (where FC represents fold change) and *P* value were 1 and 0.05, respectively. Continuous variables were expressed as the mean ± standard deviation (SD) and analyzed using two‐tailed Student’s *t*‐test; *P* < 0.05 was considered significantly different.

## Results

### Overexpression of IL13RA2 in HCC predicts good long‐term survival

**Figure 1:**
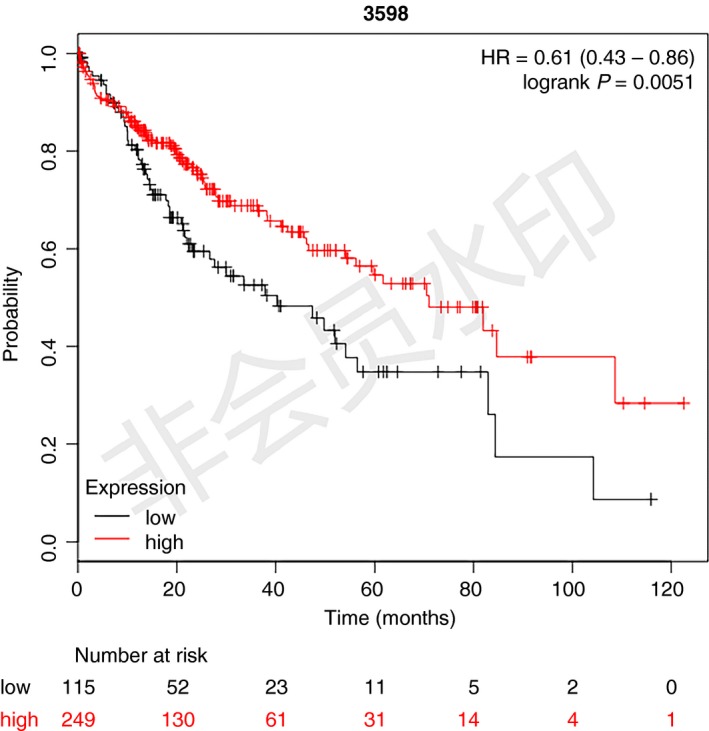
Low expression of IL13RA2 correlates with poor prognosis in patients with HCC. Kaplan–Meier plot of the correlation between IL13RA2 expression and OS, using TCGA, GEO: http://www.ncbi.nlm.nih.gov/geo/query/acc.cgi?acc=GSE20017 and GEO: http://www.ncbi.nlm.nih.gov/geo/query/acc.cgi?acc=GSE9843 integrated databases. The OS of the IL13RA2 high‐expression group was better than that of the IL13RA2 low‐expression group (*P* = 0.0051).

To identify the clinical significance of IL13RA2 in HCC, we performed a Kaplan–Meier survival analysis in Kaplan–Meier Plotter (http://kmplot.com/analysis/index.php?p=service%26cancer=liver_rnaseq), which integrated three independent transcriptomic datasets: TCGA, Gene Expression Omnibus (GEO): http://www.ncbi.nlm.nih.gov/geo/query/acc.cgi?acc=GSE20017 and GEO: http://www.ncbi.nlm.nih.gov/geo/query/acc.cgi?acc=GSE9843 23. OS available data included 364 patients. OS for patients with low IL13RA2 expression (115 patients) was significantly poorer than that for patients with high IL13RA2 expression (249 patients) (Fig. 1). Intriguingly, the clinical impact of IL13RA2 in HCC is opposite to other solid tumors.

### The expression of IL13RA2 in HCC

**Figure 2:**
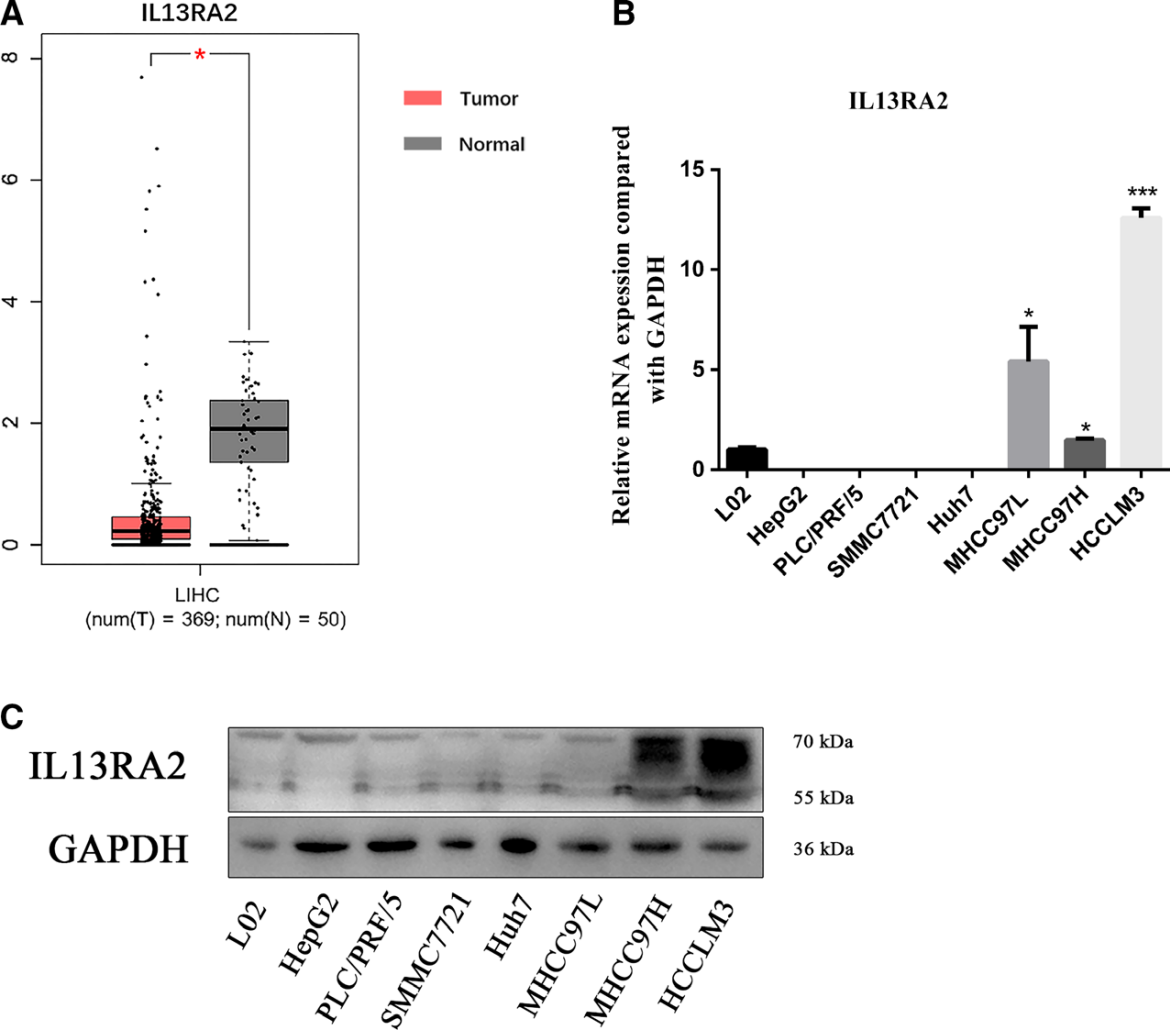
The expression of IL13RA2 in HCC. (A) TCGA analysis of IL13RA2 expression in human HCC and normal hepatic tissue. IL13RA2 expressed higher in normal hepatic tissue. (B) Quantitative RT‐PCR analysis of IL13RA2 mRNA expression in HCC cell lines and normal hepatocyte cell line (L02). The IL13RA2 mRNA expressed higher in MHCC97L cells, MHCC97H cells and HCCLM3 cells. (C) Western blot analysis of IL13RA2 protein expression in HCC cell lines and normal hepatocyte cell line (L02). The IL13RA2 expressed higher in MHCC97H cells and HCCLM3 cells. *P < 0.05; ***P < 0.001 by Student’s *t*‐test. Error bars represent SD. *n* ≥ 3 independent experiments per condition.

To determine IL13RA2 expression in human HCC and cell lines, we performed TCGA database analysis, which included 50 normal hepatic tissue samples and 369 tumor samples. TCGA database analysis revealed that the expression of IL13RA2 was higher in normal hepatic tissue compared with its expression in tumor (Fig. 2A). Then we validated IL13RA2 expression in different human HCC cell lines and normal hepatocyte (L02) via quantitative RT‐PCR and western blot. Compared with most HCC cell lines (Hep G2, PLC/PRF/5, SMMC7721, Huh7), the mRNA expression of IL13RA2 was slightly higher in hepatocyte L02 (Fig. 2B,C), which was analogous to TCGA analysis result. Intriguingly, IL13RA2 was overexpressed significantly in MHCC97 cell lines by mRNA level and highly expressed in MHCC97H and HCCLM3 cells, but not MHCC97L, by protein level (Fig. 2B,C).

### IL13RA2 silencing promotes cell proliferation and migration, but inhibits cell apoptosis

**Figure 3:**
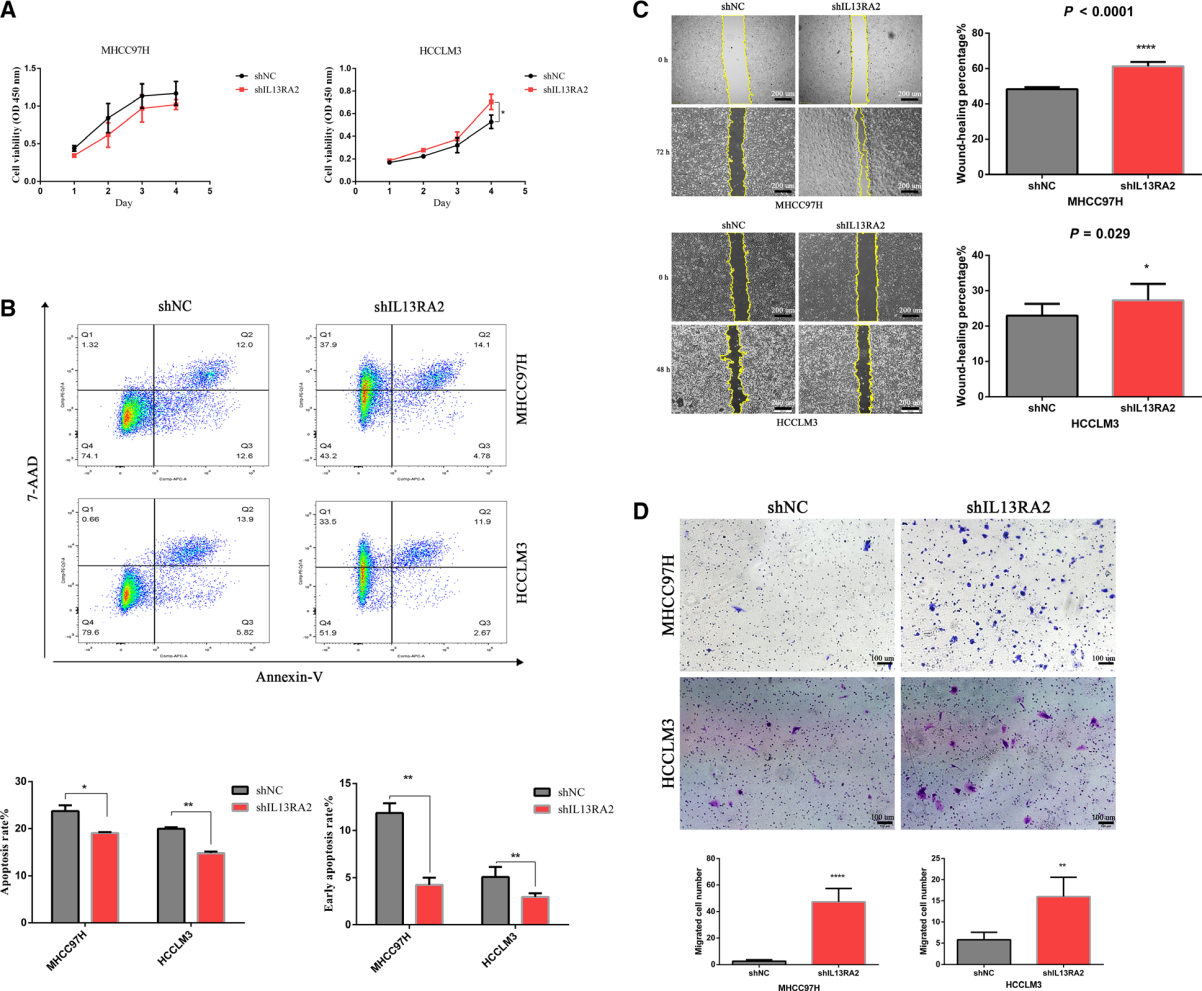
IL13RA2 silencing enhances cell proliferation and migration, but inhibits cell apoptosis. (A) CCK‐8 assay for cell proliferation of MHCC97H‐shIL13RA2 cells and HCCLM3‐shIL13RA2 cells compared with their vector control. IL13RA2 knockdown increased cell proliferation of HCCLM3, but not of MHCC97H. (B) Flow cytometry analysis of apoptosis of MHCC97H‐shIL13RA2 cells and HCCLM3‐shIL13RA2 cells compared with their vector control. IL13RA2 knockdown decreased the total and early apoptosis rates of MHCC97H and HCCLM3 cells. (C, D) Wound‐healing assays (original magnification ×40; scale bars represent 200 µm) (C) and Transwell migration assays (original magnification ×100; scale bars represent 100 µm) (D) for cell migration in the indicated group. IL13RA2 knockdown showed obvious promotion of migration abilities in MHCC97H and HCCLM3 cells. *P < 0.05; **P < 0.01; ***P < 0.001; ****P < 0.0001, by Student’s *t*‐test. Error bars represent SD. *n* ≥ 3 independent experiments per condition. 7‐AAD, 7‐aminoactinomycin D.

To characterize the role of IL13RA2 in MHCC97H and HCCLM3 cells, we knocked down IL13RA2 with stable lentivirus transfection of short hairpin RNA. As shown in Fig. 3A, IL13RA2 knockdown conferred enhanced cell proliferation in HCCLM3, whereas the change was not evident in MHCC97H cells. In addition, IL13RA2 silencing in both MHCC97H and HCCLM3 caused lower cell apoptosis rate (Fig. 3B). To determine the effect of IL13RA2 on cell migration, we used wound‐healing assays and Transwell cell migration assays. The results confirmed that IL13RA2 knockdown increased cell migration ability in HCC cells (Fig. 3C,D).

### IL13RA2 silencing enhances epithelial‐mesenchymal transition through the ERK pathway

**Figure 4:**
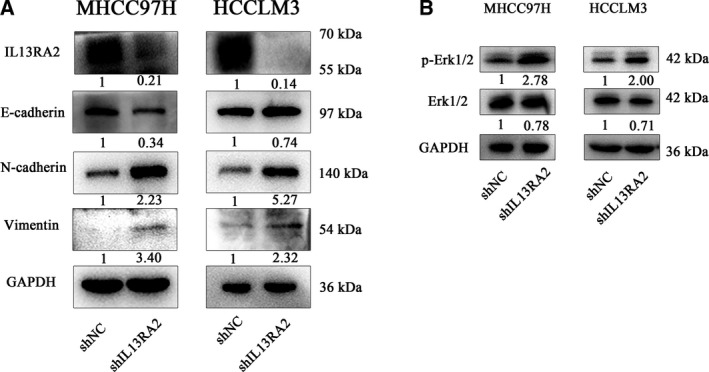
IL13RA2 knockdown induces EMT via ERK phosphorylation. (A) E‐cadherin, N‐cadherin and Vimentin were detected by western blot in MHCC97H‐shIL13RA2 cells and HCCLM3‐shIL13RA2 cells compared with their vector control. E‐cadherin was significantly decreased in the IL13RA2 knockdown group of MHCC97H cells, with N‐cadherin and Vimentin increased, whereas in the IL13RA2 knockdown group of HCCLM3 cells, N‐cadherin and Vimentin were significantly up‐regulated, with no loss of E‐cadherin. (B) Western blot detection of Erk 1/2 and p‐Erk 1/2 protein level. IL13RA2 silencing increased the expression of p‐Erk 1/2 obviously in MHCC97H and HCCLM3 cells.

To explore the potential mechanism of IL13RA2 function in HCC, we detected the level of E‐cadherin, N‐cadherin and Vimentin in IL13RA2 knockdown HCC cells comparable with the control group. As displayed in Fig. 4A, in IL13RA2 knockdown MHCC97H cells, E‐cadherin was down‐regulated, whereas N‐cadherin and Vimentin were up‐regulated significantly. In HCCLM3 cells, IL13RA2 silencing up‐regulated the expression of N‐cadherin and Vimentin, with no obvious change in E‐cadherin. These results demonstrated that IL13RA2 knockdown HCC cells underwent different types of epithelial‐mesenchymal transition (EMT) program. Next, we studied the downstream pathway involved in EMT via western blot and found that the increase in p‐Erk expression was significant for both MHCC97H and HCCLM3 cells (Fig. 4B). Collectively, IL13RA2 silencing could induce intermediate hybrid EMT states through ERK activation in different HCC cells.

## Discussion

It is well documented that IL13RA2‐targeted therapies have gained considerable effect in tumor treatment, such as IL13RA2 monoclonal antibody 24 and IL13R‐specific chimeric antigen receptor‐modified T cells 25, 26 for glioblastoma multiforme. Hence, at the very start, we intended to evaluate the role of IL13RA2 in HCC and whether these IL13RA2‐targeted interventions are applicable to HCC.

In contrast with existing research results in other tumors, our study found that low expression level of IL13RA2 in HCC predicted a shorter survival and identified IL13RA2 as being low expressed in patients with HCC through TCGA database analysis. However, human HCC cell lines MHCC97H and HCCLM3 expressed high levels of IL13RA2 specifically, characterizing the heterogeneity in the expression of IL13RA2 on HCC. Intriguingly, the MHCC97L cell line also contained a higher level IL13RA2 mRNA, but the amount of protein expression was relatively low. As we know, the mRNA abundance of a particular gene does not necessarily have a linear relationship with its translation product protein expression, mainly because there are many levels of regulation of gene expression, such as the regulation of transcriptional level, as well as post‐transcriptional regulation and translation and post‐translational regulation. Therefore, we speculated that in MHCC97L, IL13RA2 mRNA might experience degradation or interference, which could be a novel target to elevate IL13RA2 protein expression level to suppress HCC metastasis.

Our results showed that IL13RA2 could contribute to cell apoptosis and inhibiting cell proliferation and migration without addition of IL‐13. This phenomenon has also appeared in other reports 18, 20. One illustration is that serum may contain sufficient IL‐13 or other ligands. Moreover, we found that IL13RA2 silencing could promote cell proliferation of HCCLM3, but not MHCC97H. As we mentioned earlier, not only can IL13RA2 respond to IL‐13 alone, but it can also bind with other membrane proteins to play unique functions. For example, in glioblastoma multiforme 18, no significant change was observed in cell proliferation in IL13RA2 loss tumor cells with the absence of mutant EGFR (EGFRvIII). In contrast, in the presence of the EGFRvIII, IL13RA2 interacts with EGFRvIII to exert significant influence on cell proliferation. Thus, we presume that different HCC cell lines may mean distinct receptors in the cell membrane, which could bind with IL13RA2 to exhibit different biological functions. Besides, our study neglected the soluble form of IL13RA2, like most of the studies on IL13RA2 in tumors, which may play the opposite role to membrane form. Thus, the exact form of IL13RA2 protein in MHCC97H also could be the reason for the earlier result. Therefore, more about the form and synergistic membrane protein for IL13RA2 in HCC needs to be explored, which may open new viewpoints and provide novel targets for HCC therapeutics.

The latest research showed that EMT is not a binary process but a continuous process 27; that is, tumor cells will pass through complete epithelial state, transitional hybrid state and complete mesenchymal state. In other words, there are diverse EMT stages in tumor. These subpopulations have different cell plasticity, invasion and metastasis ability. Importantly, hybrid EMT has proved to indicate a higher invasion and metastasis ability 27, and E‐cadherin is required for collective cell migration and metastatic colonization 28. Hence mechanisms of partial EMT urgently need to be explored. Full EMT is regulated by clusters of transcription factors and signaling receptors. McFaline‐Figueroa *et al.* 29 found that distinct transcription factor and receptor gene knockouts could enrich a particular EMT gene profile, such as E‐cadherin, leading to a stable intermediate EMT phenotype. In our study, when IL13RA2 was silenced in MHCC97H cells, the epithelial cell marker E‐cadherin was down‐regulated dramatically, with mesenchymal cell markers N‐cadherin and Vimentin up‐regulated; whereas in HCCLM3 cells, the mesenchymal cell markers N‐cadherin and Vimentin were up‐regulated, with retained expression of epithelial cell marker E‐cadherin, which is classically characterized as partial or intermediate EMT. As we know, different HCC cell lines *in vitro* also present genetic heterogeneity, which could enable diverse partial EMT states 30. In agreement with our study, several related studies have confirmed that overexpression of Slug induces complete EMT in HepG2 cells, with partial EMT in Huh7 cells 31. In addition, transforming growth factor‐β can induce a partial EMT state in PLC/PRF/5 cells, increasing the expression of CD44, without losing epithelial cell adhesion molecule and CD133 expression, whereas in Hep3B cells, transforming growth factor‐β treatment provokes a complete EMT 32. However, the difference in transcript factors and receptors or signaling pathways between HCC cell lines leading to distinct EMT stages is yet to be understood.

ERK signaling pathway is one of the canonical pathways in tumors, playing a pivotal role in EMT 33. Our study found that HCC cells with IL13RA2 knockdown showed a high level of ERK phosphorylation, indicating that IL13RA2 may suppress EMT in HCC via inhibiting ERK activation. Thus, further investigations are required to reveal the detailed connections between IL13RA2 and the ERK signaling pathway.

## Conflict of interest

The authors declare no conflict of interest.

## Author contributions

YW conceived the project and reviewed the report. MW and RY collected and analyzed the data. MW interpreted the results and wrote the paper. All authors read and approved the final manuscript.
